# The Impact of Serum Creatinine, Albumin, Age, and Gender on the Development of Contrast-Induced Nephropathy in Patients Exposed to Contrast Agent Upon Admission to the Emergency Department

**DOI:** 10.7759/cureus.11051

**Published:** 2020-10-20

**Authors:** Canan Akman, Serkan Bakirdogen

**Affiliations:** 1 Emergency Department, Çanakkale Onsekiz Mart University Faculty of Medicine, Canakkale, TUR; 2 Nephrology Department, Çanakkale Onsekiz Mart University, Canakkale, TUR

**Keywords:** age, gender, creatinine, albumin, contrast induced nephropathy

## Abstract

Background and objectives

As the stage progresses in chronic kidney disease (CKD), the risk of contrast-induced nephropathy (CIN) also increases. Serum albumin level is the strongest predictor of CIN development in patients with CKD. It is widely known that females of age 75 are at risk for the development of CIN. Our study aims to investigate the impact of age, gender, serum creatinine, and albumin levels on the development of CIN in patients who were admitted to the emergency department and have had contrast-enhanced computerized tomography (CECT) for diagnosis.

Materials and methods

The study was planned retrospectively. Patients who applied to the emergency department between January 1, 2018, and January 1, 2020, and had CECT were included in the study. A 25% or 0.5 mg/dL increase in serum basal creatinine level within 72 hours following the implementation of contrast agent was accepted as CIN. The patients were divided into two groups: CIN (+) and CIN (-).

Results

One-hundred twenty-two patients (53 female and 69 male), whose average age was 72.27± 12, were included in the study. Forty-five of the patients were found to be CIN (+) and 77 CIN (-). There was no significant difference between the groups (p> 0.05) in terms of age. It was found that the serum creatinine level during admission to the emergency department was the determinant for the development of CIN (p = 0.024). In addition, it was observed that serum albumin levels during the admission had no impact on the development of CIN (p = 0.326). When the serum albumin values of female and male patients diagnosed with CIN measured at the first admission to the emergency service were compared, the mean values were found to be lower in male patients (p = 0.027).

Conclusion

Serum creatinine and albumin levels, age, and gender parameters should be considered in terms of the risk of CIN development in patients who are admitted to the emergency department and given contrast agents.

## Introduction

Contrast-induced nephropathy (CIN) is an acute kidney injury (AKI) that develops upon using a contrast agent. CIN is defined as an increase in the level of serum basal creatinine over 25% or 0.5 mg/dL within 72 hours of exposure to a contrast agent. A contrast agent can cause renal ischemia and direct tubulus toxicity [1-2]. CIN is the third most common cause of hospital-acquired AKI, with a frequency of 10% [3]. As the stage progresses in chronic kidney disease (CKD), the risk of CIN also increases. There are many factors that contribute to the pathogenesis of CIN [4]. The serum albumin level is the strongest predictor of CIN development in patients with CKD [5]. The serum albumin level of the patients who developed CIN after exposure to the contrast agent was found to be lower than the ones without contrast nephropathy [6]. The literature shows that the female gender is a risk factor for the development of CIN [7-8]. In addition, being over 75 years of age is an independent risk factor for the development of CIN [2]. In our study, we aimed to investigate the impact of the parameters of age, gender, serum creatinine, and albumin levels on the development of CIN in patients admitted to the emergency department and exposed to a contrast agent.

## Materials and methods

Our study was planned retrospectively. After the approval of the Local Ethical Committee (Reference number: 2020-09), among 941 patients who applied to the emergency service of the Health Practice and Research Hospital between January 1, 2018, and January 1, 2020, 122 patients aged 18 and over who had contrast-enhanced computerized tomography (CECT) were included in the study. The exclusion criteria of the study were determined as follows: being under the age of 18, the diagnosis of chronic renal failure, hemodialysis or peritoneal dialysis, serum creatinine level above 4 mg/dL, and incomplete medical records of the patient. A 25% or 0.5 mg/dL increase in serum basal creatinine level within 72 hours following the implementation of a contrast agent was accepted as CIN [1-2]. The patients were divided into two groups: CIN (+) and CIN (-).

The serum creatinine, urea, albumin, calcium, and uric acid levels of the patients were analyzed using the colorimetric method in the Roche Cobas 6000-501 module (Roche Diagnostics, Basel, Switzerland). An ion-selective electrode (ISE) and potentiometric method were used in the analysis of ionized calcium in the biochemistry laboratory using Radiometer ABL 800 (Copenhagen, Denmark).

Since the biochemistry data of patients with and without CIN and patients with different gender did not show normal distribution (p <0.05), they were compared using the Mann-Whitney U test. When the patients were admitted to the emergency service, their serum creatinine, urea, albumin, uric acid, calcium, and ionized calcium levels were determined. The same levels were reevaluated 72 hours after contrast agent administration. Therefore, the levels of the groups with and without CIN at the time of admission to the emergency department and after 72 hours were tested using the Wilcoxon signed-rank test. The ages of the groups with and without nephropathy were compared after contrast agent administration. Since the ages showed a normal distribution, it was compared using the student's t-test. The distribution of patients with and without CIN after contrast agent according to the gender variable was compared using the chi-square test. Multinomial logistic regression was applied to determine the impact of biochemistry results on gender groups and on whether diagnosed with CIN or not. p <0.05 was used for statistical significance. The Statistical Package for the Social Sciences (SPSS) version 19.0 (IBM Corp, Armonk, NY) was used in the analysis.

## Results

One hundred twenty-two patients (53 female and 69 male) were included in the study. The average age of the patients was found to be 72.27 ± 12. After CECT, 45 patients were determined as CIN (+) and 77 as CIN (-). CIN developed in 45 patients (13 female and 32 male). There was no significant difference between the groups in terms of age (p > 0.05). However, a significant difference was found in terms of gender (p = 0.013). In the examinations performed after contrast agent administration, serum creatinine, urea, albumin, uric acid, calcium, and ionized calcium levels were compared in CIN (+) and CIN (-) groups during admission to the emergency department and 72 hours after contrast agent were administered. The results are shown in Table 1. When the patients included in the study were evaluated according to their comorbidity, hypertension (n = 29), malignancy (n = 25), and coronary artery disease (n = 9) were the top three among the illnesses. A comparison of the patient groups in terms of age is shown in Table 2 and the comparison of gender distributions in Table 3. Gender, diagnosis of CIN after contrast agent administration, first admission to the emergency room, and biochemistry results obtained at 72 hours after contrast agent were analyzed with comparison tests. Multinomial logistic regression was applied using variables with significant results in these comparison tests. In the regression analysis, the male patient group with CIN was taken as the reference. The comparisons were interpreted according to this group. Being a male and having a high serum creatinine level increased the risk of being diagnosed with CIN approximately one-fold as compared to being a female patient and having a low serum creatinine level (Exp [B] = 1.148). Similarly, being a female and being older increased the risk of being diagnosed with CIN approximately one-fold as compared to being male and being at a younger age (Exp [B] = 1.113). Furthermore, being a female patient and having a high pre-contrast serum albumin level increased the risk of being diagnosed with CIN nearly four times as compared to being a male and having a low pre-contrast serum albumin level (Exp [B] = 4.362). Multinomial logistic regression analysis in patient groups is given in Table 4. Chi-square test results showed that there was a difference in the gender distribution of CIN (+) and CIN (-) patients after contrast agent administration (p <0.05). This difference was obtained at a small effect size (ɸc = 0.22). In the chi-square test, the differences between the groups were compared. The percentage of female patients is higher in the CIN (-) group, whereas the percentage of males in the CIN (+) group is higher. It was found that the serum creatinine level at the time of admission was a determinant of the development of CIN (p = 0.024). It was also found that serum urea, albumin, uric acid, corrected calcium and ionized calcium levels during admission to the emergency department had no impact on the development of CIN (p> 0.05). The comparison of serum albumin and creatinine levels between the CIN (+) and CIN (-) groups before contrast agent administration is shown in Table 5 and the comparison of serum albumin levels measured at the first admission to the emergency service of female and male patients diagnosed with CIN is given in Table 6.

**Table 1:** Comparison of the biochemistry results of the patient groups before (I) and after (II) contrast agent CIN: contrast-induced nephropathy

Group	Variable	Mean_ I_(Standard Deviation)_I_ / Mean _II_ (Standard Deviation)_II_	Med_I_ (Min-Max) / Med_ II_ (Min-Max)	p
CIN (-) (N=77)	Creatinine (mg/dL)	0.95±0.31 / 0.79±0.28	0.93 (0.38-1.65) /0.74 (0.28-1.46)	<0.0001
	Urea (mg/dL)	45.24±23.08 / 39.69±23.69	40.0(10.6-139.0)/34.4 (2.8-138.1)	<0.0001
	Uric acid (mg/dL)	5.90±2.18 / 5.37±2.33	5.9(2.2-13.2) / 4.8(2.1-15.4)	0.004
	Corrected Calcium (mg/dL)	9.23±0.84 / 9.20±0.75	9.31(5.8-13.4) / 9.14(7.8-14.1)	0.400
	Ionized calcium (mmol/L)	1.13±0.08 / 1.11±0.08	1.12(0.87-1.58)/ 1.12(0.83-1.33)	0.076
	Albumin (g/dL)	3.60±0.68 / 3.13±0.62	3.63(1.81-5.2) / 3.10(1.55-4.65)	<0.0001
CIN (+) (N=45)	Creatinine (mg/dL)	1.15±0.48 / 1.57±0.85	1.04(0.48-3.09) / 1.39(0.63-4.79)	<0.0001
	Urea (mg/dL)	53.4±28.9 / 77.7±46.8	49.5(8.30-179.4) / 66.6(20.2-229.0)	<0.0001
	Uric acid (mg/dL)	5.85±2.43 / 6.15± 2.73	5.20(2.0-11.6) / 5.50(2.20-13.40)	0.112
	Corrected calcium (mg/dL)	9.38±0.59 / 9.25±0.75	9.48(7.77-10.97) / 9.27(7.03-12.27)	0.138
	Ionized calcium (mmol/L)	1.13±0.08 / 1.10±0.10	1.13(0.96-1.38) / 1.10(0.86-1.40)	0.123
	Albumin (g/dL)	3.44±0.75 / 3.17±0.72	3.52(1.61-5.19) / 3.27(1.56-4.34)	0.002

**Table 2:** Comparison of the age distributions of the patient groups CIN: contrast-induced nephropathy

Group	Mean (SD)	p
CIN (-)	71.10±11.83	0.161
CIN (+)	74.27±12.19	

**Table 3:** Comparison of the gender distributions of patient groups CIN: contrast-induced nephropathy

		Gender		Total	p	Effect Size (ɸ_c_)
		Female	Male			
CIN (-)	F	40	37	77	0.013	0.22
	% Contrast-induced nephropathy	51.9	48.1	100		
	% Gender	75.5	53.6	63.1		
CIN (+)	F	13	32	45		
	% Contrast-induced nephropathy	28.9	71.1	100		
	% Gender	24.5	46.4	36.9		

**Table 4:** The impact of biochemistry results on CIN (+) and (-) gender groups (multinomial logistic regression) CIN: contrast-induced nephropathy

Groups/Variables		B	Wald	Sig.	Exp (B)	95% Confidence Interval for Exp (B)	
Female Without CIN	Intercept	12.213	0.000	0.992			
	Age	0.353	0.001	0.973	1.423	2.492	812353483.010
	Pre creatinine	462.968	0.181	0.670	1.160	0.000	
	Pre albumin	6.035	0.000	0.987	417.705	0.000	
	Post creatinine	-511.036	60539.099	0.000	1.148	1.959	6.729
	Post urea	0.127	0.000	0.986	1.135	7.662	1681708.580
Male Without CIN	Intercept	12.136	0.000	0.992			
	Age	0.302	0.001	0.977	1.352	2.369	772198455.742
	Pre creatinine	462.332	0.181	0.671	6.140	0.000	
	Pre albumin	6.462	0.000	0.986	640.549	0.000	
	Post creatinine	-508.213	.	.	1.932	1.932	1.932
	Post urea	0.138	0.000	0.985	1.147	7.745	1699943.204
Female + CIN	Intercept	-13.682	7.134	0.008			
	Age	0.107	5.209	0.022	1.113	1.015	1.221
	Pre creatinine	-0.515	.196	0.658	0.597	0.061	5.858
	Pre albumin	1.473	3.997	0.046	4.362	1.029	18.485
	Post creatinine	-0.053	.005	0.942	0.948	0.227	3.956
	Post urea	-0.001	.008	0.928	0.999	0.973	1.025

**Table 5:** Comparison of serum albumin and creatinine levels between the CIN (+) and CIN (-) groups before the administration of contrast agent CIN: contrast-induced nephropathy

	State of Diagnosis Upon Contrast	N	Mean (SD)	Median (Min-Max)	p
Creatinine (mg/dL)	Without CIN	77	0.95 (0.31)	0.93 (0.38-1.65)	0.024
	With CIN	45	1.15 (0.48)	1.04 (0.48-3.09)	
Albumin (g/dL)	Without CIN	77	3.60 (0.68)	3.63 (1.81-5.20)	0.326
	With CIN	45	3.44 (0.75)	3.52 (1.61-5.19)	

**Table 6:** Comparison of the serum albumin levels of female and male patients diagnosed with CIN at the first admission to the emergency department CIN: contrast-induced nephropathy

	Gender	N	Mean (SD)	Median (Min-Max)	p
Albumin (g/dL) Level	Female	13	3.82 (0.66)	3.94 (2.31-5.19)	0.027
	Male	32	3.28 (0.74)	3.33 (1.61-4.38)	

## Discussion

The risk of CIN increases as the stage progresses in CKD [4]. As the glomerular filtration rate (GFR) drops below 60 mL/min/1.73 m², the incidence of CIN increases [9]. In our study, we observed that the average serum creatinine level of the patients with CIN (+) was higher than that of patients with CIN (-). The serum creatinine levels of patients with CIN (+) at the 72^nd^ hour were higher than the levels at admission to the emergency department, and there was a significant difference between the levels. The fact that the serum creatinine levels at the 72^nd^ hour in patients with CIN (-) are lower than the levels at the time of admission may be due to the parenteral fluid support administered in the emergency service.

The serum albumin level is the strongest predictor of the development of CIN in patients with CKD [5]. The serum albumin level of the patients who developed CIN after exposure to a contrast agent was found to be lower than the level of the patients without CIN [6]. In our study, we observed that the serum albumin level of the patients during their admission to the emergency department did not have an impact on the development of CIN. The serum albumin level was found to be lower in the patient group with CIN (+) than in the group with CIN (-). The average serum albumin level was found to be higher in females than males at the time of admission to the emergency department. In experimental studies, vacuolation is experienced in proximal tubular epithelial cells in the initial stages of CIN development, and this situation is more significant in females than males [10]. It is stated that being female is a risk factor for the development of CIN [7-8]. In the study, there was a significant difference between the groups in terms of gender distribution. The proportion of female patients was higher in the CIN (-) group, whereas the number of male patients was higher than the number of female patients in the CIN (+) group. Increasing serum creatinine levels in CKD increases the risk of developing CIN [4]. It was found in our study that being a male and having a high serum creatinine level increased the risk of being diagnosed with CIN approximately one-fold as compared to being a female patient and having a low serum creatinine level. This may be due to the high serum creatinine levels and because the patients CIN (+) were mostly male.

Mehran et al. stated that age over 75 is an independent and determinant factor of the development of CIN [2]. Although there was no statistically significant difference between the two groups in terms of age in our study, the average age of the patients with CIN (+) was found to be 74.27 ± 12.19. Being a female and being older increase the risk of being diagnosed with CIN approximately one-fold as compared to the males and being younger. This finding correlates with the related research in the literature [2,8].

## Conclusions

In conclusion, in addition to serum creatinine and albumin levels, age and gender parameters should also be considered in terms of the risk of CIN development in the patients admitted to the emergency department and given a contrast agent. To summarize: (a) Being a male patient and having a high serum creatinine level increases the risk of being diagnosed with CIN approximately one-fold as compared to being a female patient and having a low serum creatinine level (Exp [B] = 1.148); (b) Being a female patient and being older increases the risk of being diagnosed with CIN approximately one-fold as compared to being male and being at a younger age (Exp [B] = 1.113); (c) Being a female patient and having a high pre-contrast serum albumin level increases the risk of being diagnosed with CIN nearly four times as compared to being a male and having a low pre-contrast serum albumin level (Exp [B] = 4.362); (d) Serum creatinine levels at the admission stage are determinant of the development of CIN; (e) Serum albumin levels at the time of admission to the emergency department have no impact on the development of CIN.
